# Identification of the I38T PA Substitution as a Resistance Marker for Next-Generation Influenza Virus Endonuclease Inhibitors

**DOI:** 10.1128/mBio.00430-18

**Published:** 2018-04-24

**Authors:** Jeremy C. Jones, Gyanendra Kumar, Subrata Barman, Isabel Najera, Stephen W. White, Richard J. Webby, Elena A. Govorkova

**Affiliations:** a Department of Infectious Diseases, St. Jude Children’s Research Hospital, Memphis, Tennessee, USA; b Department of Structural Biology, St. Jude Children’s Research Hospital, Memphis, Tennessee, USA; c Independent consultant and collaborator with St. Jude Children’s Research Hospital, Memphis, Tennessee, USA; NIAID, NIH

**Keywords:** PA polymerase, antiviral, antiviral resistance, endonuclease, influenza virus

## Abstract

The clinical severity and annual occurrence of influenza virus epidemics, combined with the availability of just a single class of antivirals to treat infections, underscores the urgent need to develop new anti-influenza drugs. The endonuclease activity within the viral acidic polymerase (PA) protein is an attractive target for drug discovery due to the critical role it plays in viral gene transcription. RO-7 is a next-generation PA endonuclease inhibitor of influenza A and B viruses, but its drug resistance potential is unknown. Through serial passage of influenza A(H1N1) viruses in MDCK cells under selective pressure of RO-7, we identified an I38T substitution within the PA endonuclease domain that conferred *in vitro* resistance to RO-7 (up to a 287-fold change in 50% effective concentration [EC_50_]). I38T emerged between 5 and 10 passages, and when introduced into recombinant influenza A(H1N1) viruses, alone conferred RO-7 resistance (up to an 81-fold change in EC_50_). Cocrystal structures of mutant and wild-type endonuclease domains with RO-7 provided the structural basis of resistance, where a key hydrophobic interaction between RO-7 and the Ile38 side chain is compromised when mutated to the polar threonine. While Ile38 does not have a crucial role in coordinating the endonuclease active site, the switch to threonine does affect the polymerase activity of some viruses and influences RO-7 affinity for the PA_N_ target (i.e., the ≈200-residue N-terminal domain of PA). However, the change does not lead to a complete loss of replication activity *in vitro*. Our results predict that RO-7-resistant influenza viruses carrying the I38T substitution may emerge under treatment. This should be taken into consideration for clinical surveillance and in refinement of these drugs.

## IMPORTANCE

The effectiveness of antiviral drugs can be severely compromised by the emergence of resistant viruses. Therefore, determination of the mechanisms by which viruses become resistant is critical for drug development and clinical use. RO-7 is a compound that potently inhibits influenza virus replication and belongs to a new class of drugs in late-stage clinical trials for treatment of influenza virus infection. Here we demonstrate that a single amino acid change acquired under prolonged virus exposure to RO-7 renders influenza viruses significantly less susceptible to its inhibitory effects. We have discovered how the mutation can simultaneously interfere with drug activity and still maintain efficient virus replication. These findings have important implications for the development of more effective derivatives of RO-7-like drugs and provide guidance for how to monitor the emergence of resistance.

## OBSERVATION

Despite the availability of vaccines, influenza viruses continue to circulate in humans and cause disease. Both seasonal influenza virus infection and infections caused by zoonotic subtypes (i.e., H5N1, H7N9, and H10N8) are significant public health burdens (1). Antiviral therapies are important for the prevention and treatment of influenza, but they are currently limited to a single class of drugs, namely, the neuraminidase inhibitors (NAIs). The rise of biologically fit, NAI-resistant influenza A(H1N1) viruses in 2007 through 2009 (2, 3), together with the overreliance on NAI monotherapy, highlights the urgent need to develop new anti-influenza inhibitors with diverse viral protein targets and novel mechanisms of action.

The influenza virus RNA-dependent RNA polymerase (RdRp) is a trimeric complex with multiple active sites and functionalities. It has recently emerged as an important therapeutic focus for drug discovery (4). The acidic polymerase (PA) protein component of the RdRp plays a critical role in viral gene transcription by mediating the process by which the polymerase performs “cap snatching” from host messenger mRNAs. The RdRp endonuclease activity that is necessary for this to occur is located within the ≈200-residue N-terminal domain of PA (PA_N_). It is widely recognized as a promising target for new influenza antivirals (5, 6).

Previous studies have examined the resistance potential of L-742,001, a first-generation 2-substituted-4,5-dihydroxypyrimidine derivative PA endonuclease inhibitor. Serial passage of A/Puerto Rico/8/1934 (H1N1) (PR/8) in the presence of L-742,001 yielded the PA-T20A mutant that increased the 50% effective concentration (EC_50_) >2-fold (7). A random mutagenesis approach in the PR/8 or A/California/04/2009 (H1N1)pdm09 (CA/04) background identified PA-T20A, -I79L, -F105S, and -E119D mutants that increased the EC_50_ up to 11-fold (8). These residues all cluster around the PA endonuclease active site. L-742,001-resistant viruses retained catalytic activity and fitness (8). Other studies have introduced strategic mutations (at residues 20, 24, 37, 38, 84, 122, and 130) within the PR/8 PA endonuclease domain, some of which also had negligible impact upon polymerase activity but increased virus resistance to L-742,001 (9). The recent availability of high-quality structural data for the PA protein (5, 6) has allowed for structure-based drug design of next-generation endonuclease inhibitors with enhanced EC_50_s compared to L-742,001-like compounds. To date, two drugs have advanced into clinical trials: S-033188/baloxavir marboxil (Shionogi and Co., Ltd., Japan) and AL-794/JNJ64155806 (Alios BioPharma, USA; Janssen Pharmaceutica, Belgium) (4), but no resistance profiles for these drugs have thus far been published. The development pipeline for these and other influenza virus PA inhibitors must include analysis of resistance potential. Such information can inform dosing in clinical settings, guide surveillance of resistance among circulating viruses and in treated patients, and direct drug design to avoid interactions with resistance-prone residues.

We recently demonstrated that a novel PA inhibitor, RO-7, displays broad-spectrum anti-influenza activity *in vitro* (10) and protects mice from lethal challenge with both influenza A and B viruses (11). Although no resistant viruses were identified from the lungs of RO-7-treated, virus-infected mice, the potential for antiviral resistance to emerge with extended RO-7 pressure is unknown. Here, we report an analysis of the resistance potential to RO-7. We serially passaged two influenza A viruses, CA/04 and PR/8, in MDCK cells 16 times in the presence of increasing concentrations of RO-7 (from 6 nM to 1 µM [P1 to P16]) followed by 5 additional passages in the absence of drug (S1 to S5) to assess the stability of any developed resistance (Table 1).

**TABLE 1:** Genotypic and phenotypic characteristics of RO-7-resistant influenza A(H1N1) viruses selected in MDCK cells

Virus and passage type	Passage no.	RO-7 concn (nM)	PA genotype^a^	Degree of inhibition by virus yield reduction assay (log_10_ TCID_50_/ml)^b^			Plaque reduction assay		Minireplicon assay	
				10 nM	100 nM	1,000 nM	EC_50_ (nM)^c^	Fold change	EC_50_ (nM)^d^	Fold change
A/California/04/2009 (H1N1)pdm09										
RO-7 serial passage	0	0	Ile	7.7 ± 0.1	7.7 ± 0.1	7.7 ± 0.1	3 ± 1	1	−^e^	−
	3	6	Ile	7.5 ± 0.3	7.5 ± 0.3	7.5 ± 0.3	9 ± 3	3	−	−
	5	18	Ile	7.5 ± 0.3	7.5 ± 0.3	7.5 ± 0.3	−	−	−	−
	10	162	Thr	1.6 ± 1.4	1.0	1.0	671 ± 144	224	−	−
	16	1000	Thr	0.5 ± 0.5	0.3 ± 0.4	<^f^	538 ± 231	179	−	−
Mock passage	16	0	Ile	7.8 ± 0.5	7.8 ± 0.5	7.8 ± 0.5	6 ± 3	2	−	−
Mutant stability	S5^g^	0	Thr	0.5 ± 0.7	<	0.2 ± 0.7	860 ± 93	287	−	−
rg-CA/04-WT^h^	−	−	Ile	4.3 ± 1.6	5.1 ± 0.3	5.1 ± 0.3	3 ± 0.3	−	11 ± 2	−
rg-CA/04-I38T^h^	−	−	Thr	<	<	3.5 ± 0.3	227 ± 119	76	599 ± 97	54

A/Puerto Rico/8/1934 (H1N1)										
RO-7 serial passage	0	0	Ile	7.6 ± 0.2	7.6 ± 0.2	7.6 ± 0.2	3 ± 1	1	−	−
	3	6	Ile	7.1 ± 0.5	7.1 ± 0.5	7.1 ± 0.5	19 ± 7	6	−	−
	5	18	Thr	0.8 ± 0.7	0.6 ± 0.4	7.5 ± 0.3	−	−	−	−
	10	162	Thr	<	<	1.1 ± 0.8	365 ± 188	122	−	−
	16	1000	Thr	0.3 ± 1.0	<	<	867 ± 84	289	−	−
Mock passage	16	0	Ile	7.8 ± 0.4	7.8 ± 0.4	7.8 ± 0.4	2 ± 1	−	−	−
Mutant stability	S5^g^	0	Thr	0.4 ± 0.6	0.3 ± 0.4	0.4 ± 0.2	564 ± 97	188	−	−
rg-PR/8-WT^h^	−	−	Ile	2.7 ± 0.6	7.6 ± 0.6	7.6 ± 0.6	4 ± 1	−	16 ± 1	−
rg-PR/8-I38T^h^	−	−	Thr	<	2.0 ± 1.0	3.5 ± 0.4	322 ± 217	81	513 ± 22	32

a Amino acid identity at residue 38 of the PA protein as determined by Sanger sequencing.

b Reduction of virus yield (log_10_) from infected MDCK cells (MOI of 0.01; *n =* 2 wells/drug concentration/virus) at 72 hpi as determined by titration in MDCK cells. Average values from 3 independent experiments are presented ± standard deviation (SD).

c Reduction of plaque formation number from infected MDCK cells (50 to 100 PFU/well; *n =* 3 wells/drug concentration/virus) at 72 hpi. Average values from 3 to 6 independent experiments are presented ± standard error of the mean (SEM).

d Reduction of luciferase reporter-generated polymerase complex activity at 24 hpi. Average values from 4 independent experiments are presented ± SEM.

e −, not performed or not applicable.

f <, the titers used to calculate the fold change were below the assay limit of detection (0.75 log_10_ TCID_50_/ml).

g Virus containing PA with a single I38T substitution was passaged 5 times in the absence of RO-7 (0 nM) to determine genotypic and phenotypic stability (S).

h Reverse-genetics (rg)-derived virus containing either wild-type (WT) PA or PA with the I38T substitution.

Sequence analysis of the PA_N_ domains after P0, P1, P3, P5, P10, P16, and S5 passages revealed the selection of an I38T substitution at P10 in CA/04 and at P5 in PR/8. Viruses with only the I38T substitution maintained high levels of replication throughout the passage scheme. However, in replicate assays that showed reduced viral replication capacity upon passage (CA/04 at P15 and PR/8 at P4) (data not shown), viruses were isolated that contained the I38T substitution in conjunction with an E23K or E31K PA substitution. The wild-type (WT) Ile38 residue was retained through 16 passages in the absence of drug (Table 1). P0 and P16 mock-passaged viruses remained highly susceptible to RO-7, with virus yield lowered up to 7 log_10_ tissue culture infective doses (TCID_50_)/ml and a plaque reduction EC_50_ of ≈3 nM. Acquisition of I38T affected the ability of RO-7 to inhibit virus yield (titers were lowered only by ≤1 log_10_ TCID_50_/ml) and increased plaque reduction EC_50_s to 538 and 867 nM in CA/04 and PR/8, respectively. Moreover, the I38T substitution was stably maintained in both viruses after 5 additional passages without drug pressure (EC_50_ range, 564 to 860 nM).

To confirm that the I38T substitution conferred the RO-7 resistance phenotype, we conducted minireplicon polymerase assays (6) in the presence of plasmids expressing either the WT (Ile38) or mutant (Thr38) residue in PA_N_. Polymerase complexes containing WT PA_N_ were highly susceptible to RO-7 inhibition, with EC_50 s_ of 11 and 16 nM in CA/04 and PR/8, respectively. In contrast, complexes containing Thr38 PA_N_ were clearly resistant, with EC_50_s changing by 54- or 32-fold with both viruses (Table 1). The I38T substitution had variable effects on virus fitness in the minireplicon assay. I38T substitution in PR/8 reactions increased polymerase activity by 43% (see Fig. S1 in the supplemental material), as reported previously (9). However, the PR/8 reverse-genetics backbone system is an optimized laboratory system for generation of vaccine stocks, and it does not necessarily represent a naturally circulating influenza virus. Therefore, we analyzed the effect of I38T with CA/04 and found that this substitution decreased polymerase activity by 48% (Fig. S1). Control reactions demonstrated that CA/04 or PR/8 WT PA_N_ reactions were highly susceptible to RO-7, while the mutant reactions were resistant (Fig. S1). Next, we generated reverse-genetics CA/04 or PR/8 virus variants (rg-CA/04 and rg-PR/8) containing either WT or mutant PA. In plaque reduction assays, rg-CA/04-WT and rg-PR/8-WT viruses were susceptible to RO-7 (EC_50_, 3 to 4 nM), similar to the WT viruses. rg-CA/04-I38T and rg-PR/8-I38T viruses displayed significantly elevated EC_50_s (227 and 322 nM), confirming I38T as a mediator of RO-7 resistance (Table 1).

10.1128/mBio.00430-18.1 FIG S1  Impact of the I38T PA substitution on polymerase activity. Polymerase activities of CA/04 or PR/8 influenza virus polymerase complexes were determined by minireplicon assay in HEK293T cells transfected with plasmids expressing viral proteins NP, PB1, and PB2, with a luciferase and β-galactosidase reporter. Either the WT (Ile38) or the mutant (Thr38) PA plasmid was also cotransfected. Control reactions were treated with RO-7 (250 nM) 3 h before and 24 h after transfection. The polymerase activity was measured relative to that in the untreated control (0 nM RO-7 [mock]) as luciferase activity normalized to β-galactosidase activity. Data are presented as the combined mean values (triplicate or quadruplicate replicates) ± SD from 4 independent assays. *, *P* ≤ 0.05; ****, *P* ≤ 0.0001. Download FIG S1, TIF file, 0.2 MB. Copyright © 2018 Jones et al. 2018 Jones et al. This content is distributed under the terms of the Creative Commons Attribution 4.0 International license.

We hypothesized that RO-7 resistance profiles may be due to the I38T substitution lowering the affinity of PA_N_ for the compound, and this was confirmed using isothermal titration calorimetry (ITC). Ile PA_N_ binds RO-7 with a *K*_*D*_ (equilibrium dissociation constant) of 9.5 nM, whereas Thr38 PA_N_ binds RO-7 with a *K*_*D*_ of 4.6 µM (see Fig. S2 in the supplemental material). Thus, this single mutation leads to an approximate 500-fold reduction in affinity for the inhibitor.

10.1128/mBio.00430-18.2 FIG S2  Isothermal titration calorimetry. Shown are representative binding isotherms for the interaction of RO-7 with (A) WT (Ile38) or (B) mutant (Thr38) PA_N_. The upper panel in the isotherm shows the raw heat change upon binding, and the bottom panel shows the integrated heat change associated with each injection. The data presented are representative of 3 independent assays, with *K*_*D*_ values indicative of the means ± SD of all assays. Download FIG S2, TIF file, 0.1 MB. Copyright © 2018 Jones et al. 2018 Jones et al. This content is distributed under the terms of the Creative Commons Attribution 4.0 International license.

**FIG 1:**
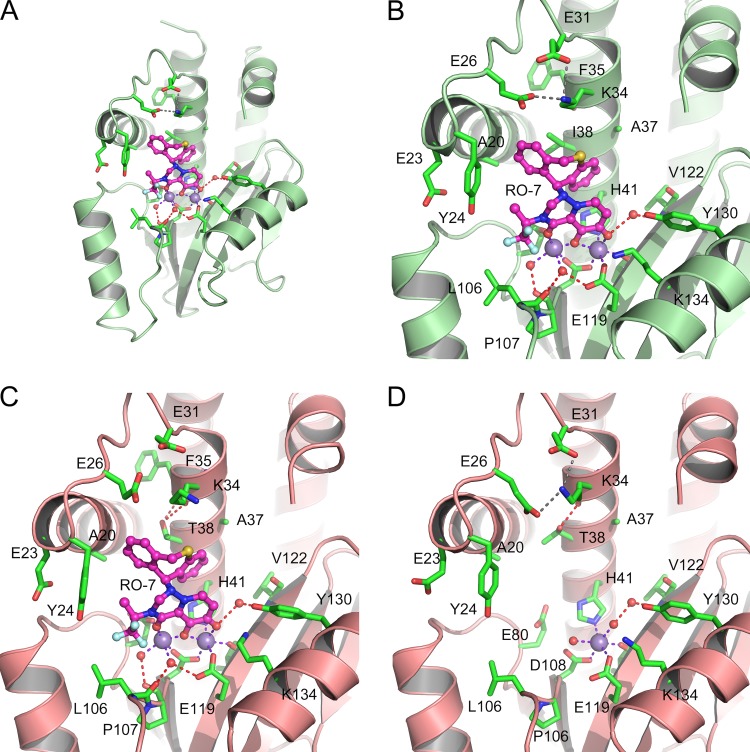
Crystal structures of WT and I38T PA_N_ in complex with RO-7. (A) Overall view of the RO-7 complex with the WT (Ile38) PA_N_ showing the binding locale in the large RNA substrate-binding cleft. (B) Close-up view of panel A with the key residues labeled. (C) Close-up view of the RO-7 complex with mutant Thr38 PA_N_. (D) Close-up view of holo-Thr38 PA_N_. The orientations of PA_N_ are identical in all four panels, and the WT and mutant proteins are shown as green and salmon ribbons, respectively, to distinguish these variants. Residues are shown as green carbon sticks, Mn^2+^ ions are violet balls, water molecules are red balls, and RO-7 is shown in the ball-and-stick representation with magenta carbon atoms. Metal coordination bonds, hydrogen bonds, and salt bridges are shown as purple, red, and gray dashed lines, respectively.

To gain structural insights into the I38T-mediated RO-7 resistance and the associated reduction in binding, we determined the crystal structures of WT and mutant PA_N_ in the presence of RO-7 at 2.09 and 2.3 Å, respectively (Table S1). Despite the significant loss in binding affinity, it was possible to obtain crystals of Thr38 PA_N_ in complex with RO-7 in the same cocrystallization conditions used for the WT complex because the RO-7 concentration (1.2 mM) was well above the measured *K*_*D*_. RO-7 binds in essentially the same way in the two structures and occupies a large pocket bounded by Tyr24 on the “left,” the Glu26-Lys34 salt bridge at the “top,” Tyr130 and Lys134 on the “right,” and Ala20, Ile38/Thr38, His41, and Glu80 at the “back” (Fig. 1A and B and C). The 5-hydro-2,3-dihydro-1*H*-pyrido-triazine-4,6-dione moiety chelates the active-site metal ions, and the fluorine atoms of the trifluoro propane group interact weakly with the flanking OH group of Tyr24 and the main-chain carbonyl oxygen atom of Leu106. In the WT complex (Fig. 1B), the side chain of Ile38 makes an extensive hydrophobic interaction with the 6,11-dihydrodibenzo[b,e]thiepine group that has the ideal shape to wrap around the side chain. In the I38T complex (Fig. 1C), the threonine side chain is oriented such that the CG2 carbon atom can maintain this hydrophobic interaction, while the hydroxyl group forms hydrogen bonds to the backbone carbonyl oxygens of Lys34 and Phe35. Importantly, the hydrophobic interaction surface with the dibenzothiepine moiety of RO-7 is significantly reduced in Thr38 compared to the WT complex. When the two complexes are compared (see Fig. S3A in the supplemental material), there is only one minor difference apart from I38T: the loop residues Tyr24/Gly25/Glu26 move by ~0.8 Å toward RO-7 in the I38T complex. To determine whether the I38T mutation has any structural effect in the absence of bound RO-7, we determined the 2.2-Å crystal structure of holo-I38T PA_N_ (Fig. 1D; see Table S1 in the supplemental material). Compared to the holo-WT structure (8, 12), there are no significant effects on the active-site locale, although one small difference is that the Mn^2+^ ion at the documented low-affinity site (13) is not present. However, it is noticeable that the side chain of Thr38 is rotated compared to the RO-7 structure, which emphasizes the importance of maximizing the hydrophobic interaction surface when the inhibitor binds.

10.1128/mBio.00430-18.3 FIG S3  Crystal structures of WT, I38T, and E119D PA_N_ in complex with RO-7. (A) The WT (Ile38 [green ribbons]) and mutant (Thr [salmon ribbons]) complex structures shown in Fig. 1B and C superimposed. (B) The WT complex in the presence of Mg^2+^. (C) The E119D complex with Mn^2+^. The orientation of PA_N_ is identical in all three figures. Protein residues are shown as sticks, Mn^2+^ as violet balls, Mg^2+^ as green balls, and water as red balls, and RO-7 is shown in the ball-and-stick representation. Metal coordination bonds, hydrogen bonds, and salt bridges are shown as purple, red, and gray dashed lines, respectively. Download FIG S3, TIF file, 27.8 MB. Copyright © 2018 Jones et al. 2018 Jones et al. This content is distributed under the terms of the Creative Commons Attribution 4.0 International license.

10.1128/mBio.00430-18.4 TABLE S1  X-ray data collection and refinement statistics I. Download TABLE S1, DOCX file, 0.1 MB. Copyright © 2018 Jones et al. 2018 Jones et al. This content is distributed under the terms of the Creative Commons Attribution 4.0 International license.

There is some uncertainty as to which metal is naturally present at the active site (13, 14), and we therefore determined the WT complex structure in the presence of Mg^2+^ at 2.3 Å (see Table S2 and Fig. S3B in the supplemental material). In addition, we previously reported that the resistance-associated PA_N_ substitution E119D emerges in response to the first-generation endonuclease inhibitor L-742,001 (8), and we therefore also determined the complex structure with this mutation at 2 Å (Table S2; Fig. S3C). Both complexes show no significant differences from the WT complex (Fig. 1A and B). In the case of the E119D substitution, the holoenzyme only has one bound metal ion at the two-metal active site, but the second metal ion is restored to the active site by the coordinating groups provided by RO-7, as seen in the E119D–L-742,001 complex (8).

10.1128/mBio.00430-18.5 TABLE S2  X-ray data collection and refinement statistics II. Download TABLE S2, DOCX file, 0.1 MB. Copyright © 2018 Jones et al. 2018 Jones et al. This content is distributed under the terms of the Creative Commons Attribution 4.0 International license.

### Conclusions.

Here, we studied the resistance potential of two A(H1N1) viruses to a promising antiviral compound, RO-7, using genotypic, phenotypic, and structural analyses. After serial passage of the two viruses in the presence of RO-7, the I38T substitution was identified within the PA endonuclease domains of both. This substitution drastically increased EC_50_s. Recombinant A(H1N1) viruses containing only the I38T substitution demonstrated that it alone can impart RO-7 resistance. However, increased EC_50_s in the passaged viruses and the retention of this mutation after 5 passages in the absence of drug imply that compensatory mutations outside the PA endonuclease domain or in other viral proteins may be present. To examine this, the full-length genomes of the P0 and P16 of mock-passaged and S5 of RO-7-passaged CA/04 and PR/8 viruses were compared. Excluding I38T, we found 3 and 7 amino acid changes in CA/04 and PR/8 viruses, respectively (see Table S3 in the supplemental material). The substitutions were present in the PB1, PB2, PA, HA, and NS proteins of RO-7-passaged viruses, but not in P0 or P16 mock-passaged viruses. The role these substitutions may play in RO-7 and endonuclease inhibitor resistance is currently unknown. Additionally, 2 PA_N_ changes were identified in nonviable replicate passages (viral RNA could be recovered, but virus titers were below detection limits), including E23K in CA/04 and E31K in PR/8 (data not shown). E31K is associated with increased replication of egg-passaged viruses (15), while the impact of E23K is unknown. However, E23K was identified in one influenza A virus-infected patient in a phase 2 clinical trial of a similar compound (S-033188/baloxavir marboxil), suggesting that it may play a role in endonuclease inhibitor resistance (T. Shishido et al., presented at the Fifth Annual ISIRV Antiviral Group Conference, Shanghai, China, 14 to 16 June 2017).

10.1128/mBio.00430-18.6 TABLE S3  Amino acid changes in RO-7-resistant and -sensitive influenza A(H1N1) viruses. Download TABLE S3, DOCX file, 0.1 MB. Copyright © 2018 Jones et al. 2018 Jones et al. This content is distributed under the terms of the Creative Commons Attribution 4.0 International license.

Our ITC data clearly show that I38T disrupts the binding of RO-7, and the structural analyses reveal that this occurs by a reduction in the size of a central hydrophobic interface. Ile38 has key van der Waal interactions with the ribose moiety of a single nucleotide bound at the active site (8, 12), and this presumably explains its high conservation. The threonine substitution should be less effective in mediating this interaction, and this is supported by our unsuccessful efforts thus far to crystallize I38T with a bound nucleotide. While this appears to affect general polymerase activity in the case of CA/04 (Fig. S1), the effect on PR/8 is unchanged or even enhanced as previously reported (9) (Fig. S1). Overall, the mutation is not lethal to virus replication as indicated by our successful passage scheme. However, we have previously observed a similar phenomenon and demonstrated that drug resistance mutations in PA_N_ have surprisingly limited effects on viral and RdRp fitness (8). Our explanation for this is that, within the intact RdRp, the mRNA substrate not only binds to the PA_N_ active site, but also to the large RNA binding cleft of PA_N_ and the cap-binding domain of the PB2 subunit. It should also be noted that the binding mode of a single nucleotide at the PA_N_ active site may not accurately represent how the full mRNA substrate binds. We suggest that the OH group of Thr38 may contribute a hydrogen bond to help stabilize the bound mRNA, which would explain why I38T is the preferred RO-7 resistance substitution that we observed at this position.

The implications of the I38T substitution on RO-7-like compound development are significant. This substitution was readily generated by 5 to 10 serial passages under drug pressure in two distinct viruses. The substitution was stable and persisted for at least 5 passages in the absence of the drug. However, RO-7-induced resistance emerges later than has been observed for the adamantanes (the first generation of anti-influenza antiviral drugs, which were clinically ineffective and not recommended for prophylaxis or treatment due to widespread resistance), where resistance occurs as early as one passage experimentally and after only a few days of antiviral therapy in patients (16–18). The rate of RO-7 resistance acquisition is more in line with that seen with the clinically used NAIs, where substitutions mediating reduced susceptibility appear at ≥6 passages in a virus subtype-dependent manner: P7 to P9 for A(H1N1)pdm09 (19), P6 for A(H5N1) (20), P8 for A(H4N2) (21), and P6 to P9 for influenza B viruses (22). Additionally, recent data from a phase 2 clinical trial with an endonuclease inhibitor (S-033188/baloxavir marboxil), structurally similar to RO-7, reported the detection of I38T/F PA_N_ substitutions in four S-033188-treated patients (I38T, *n =* 2; I38F, *n =* 2) of 300 total patients from which virus genotypes could be obtained. The E23K PA_N_ substitution was identified in one additional S-033188-treated patient (Shishido et al., Fifth Annual ISIRV Antiviral Group Conference). These changes were associated with rebound of virus titers in patients after day 6 of the study.

Among circulating seasonal human influenza isolates, Ile38 is >99% conserved, while Thr38 was present in only 1 of 4,352 viruses surveyed and only in A(H1N1)pdm09 influenza viruses (9) (Influenza Research Database). This observation is not limited to influenza A viruses. It was reported that 100% of influenza B viruses (455 viruses surveyed) contained Ile38 at this position (9). The introduction of I38T into recombinant influenza B virus (Yamagata lineage) increased resistance to endonuclease inhibitor S-033188/baloxavir marboxil by >5-fold (Shishido et al., Fifth Annual ISIRV Antiviral Group Conference). Therefore, PA_N_ position 38 is likely an important resistance marker for the wider class of RO-7-like endonuclease inhibitors and for multiple influenza virus genera. Our data suggest that screening of residue 38 in PA_N_ in clinical trials and future therapeutic applications should be strongly considered. In addition, development of novel endonuclease inhibitors should ideally avoid interactions with this residue.

An increasing body of evidence suggests that endonuclease inhibitors will be the next approved influenza antiviral for widespread use, and baloxavir marboxil was recently (February 2018) approved in Japan. Although we report emergence of drug-resistant variants under endonuclease inhibitor pressure, this in no way should preclude the use of such compounds in future clinical settings. Indeed, analyses like ours are extremely valuable because they serve to identify potential resistance markers, which can then be utilized in further development and refinement of next-generation endonuclease inhibitors.

### Methods. (i) Compound.

RO-7 was synthesized at Hoffmann-La Roche, Ltd. (Basel, Switzerland), in collaboration with WuXi AppTec (Wuhan, China), prepared as a 10 mM stock in dimethyl sulfoxide (DMSO).

### (ii) Generation of resistant mutants and virus passage.

Influenza A/California/04/2009 (H1N1)pdm09 (CA/04) and A/Puerto Rico/8/1934 (H1N1) (PR/8) viruses (St. Jude Children’s Research Hospital Repository [SJCRH]) were passaged a total of 16 times in Madin-Darby canine kidney cells (MDCKs; ATCC, Manassas, VA), and supernatants were harvested 48 to 72 h postinfection (hpi). The 50% tissue culture infectious dose (TCID_50_) was determined after each passage and used to calculate a multiplicity of infection (MOI) of 0.01 for each subsequent passage. Viruses were either mock (0 nM RO-7) or RO-7 passaged beginning at 6 nM with increasing drug pressure of 2 or 3× (i.e., 6, 18, 54, 162, 486, or 1,000 nM) every 2 to 3 passages. An additional 5 passages in MDCK cells were performed with RO-7-passaged viruses without drug pressure to ensure the stability of the acquired substitutions.

### (iii) Genotypic analysis.

Total RNA was isolated from passaged viruses (RNeasy kit; Qiagen, Valencia, CA). The full-length genomes of the P0 and P16 of mock-passaged and S5 of RO-7-passaged viruses of both the CA/04 and PR/8 types were amplified by one-step reverse transcription-PCR (Qiagen), gel extracted, Sanger sequenced (Hartwell Center for Bioinformatics, SJCRH), and analyzed by SeqMan Pro (DNASTAR, Madison, WI) and BioEdit (Ibis Biosciences, Carlsbad, CA).

### (iv) Minireplicon assay and reverse genetics.

Influenza A virus genes from CA/04 and PR/8 were cloned into pHW2000 plasmid, propagated in Escherichia coli Top 10 (Invitrogen), and purified (Qiagen). The I38T PA substitution was inserted into the plasmid using gene-specific primers and the QuikChange site-directed mutagenesis kit (Agilent, Santa Clara, CA). Plasmids encoding NP, PA, PB1, and PB2, along with a pPolI-358 NP firefly luciferase reporter gene (kindly provided by Megan Shaw, Mount Sinai School of Medicine, New York, NY) and pCMV-β-galactosidase for normalization, were transfected into HEK293T cells (ATCC, Manassas, VA) to perform the minireplicon assays. The 8-plasmid reverse-genetics system was used with WT- or PA-I38T-expressing pHW2000 plasmids to rescue viruses (23).

### (v) PA protein cloning, expression, and purification.

Sequence encoding a loop-deleted version (residues 51 to 72 replaced with a GGS linker) (14) of the PA_N_ WT or the point mutation (I38T or E119D) from CA/04 was synthesized and inserted in pET-28a(+) expression plasmid (Genescript, Piscataway, NJ), expressed in BL21(DE3) cells, and purified by affinity chromatography and gel filtration (Protein Production Facility, SJCRH).

### (vi) Phenotypic analysis.

Replication kinetics or RO-7 inhibition of virus replication in MDCKs (MOI of 0.01) was determined by virus yield (TCID_50_) or plaque reduction assays (10).

### (vii) Crystallization, X-ray data collection, and structure determination.

Proteins were concentrated to 10 mg/ml in 20 mM HEPES (pH 7.8), 200 mM NaCl, 2 mM tris(2-carboxyethyl)phosphine (TCEP), and 1 mM EDTA. Crystallization was performed using the hanging drop, vapor diffusion method using a crystallization solution that contained 0.1 M HEPES (pH 7.8) or CAPSO (*N*-cyclohexyl-2-hydroxyl-3-aminopropanesulfonic acid [pH 9.5]) buffer and 1 M ammonium sulfate as a precipitant. Other specific additives for each crystal structure are detailed in the corresponding PDB entries. For cocrystallization, 1.2 mM RO-7 was added to the crystallization solution. Crystals appeared in 2 to 4 days and were flash-frozen in liquid nitrogen after soaking in crystallization solution supplemented with 30% glycerol. X-ray diffraction data were collected at the SERCAT 22-ID or 22-BM beam lines at the Advanced Photon Source. Data sets were indexed, integrated, and scaled using HKL-2000 (24), and the structures were determined by molecular replacement using Phaser (25). Model building and refinement were done using Phenix, CCP4, and coot. For data collection and refinement statistics, see Tables S1 and S2.

### (viii) Isothermal titration calorimetry.

Thermodynamic parameters for the interaction of RO-7 with purified WT or I38T mutant PA_N_ were measured using a MicroCal auto-iTC 200 (Malvern Instruments, Malvern, United Kingdom). Protein samples were exchanged into 20 mM HEPES (pH 7.8), 100 mM NaCl, 5 mM MnCl_2_, and 0.05% Tween 20 prior to the experiment. Titrations were performed by first injecting 0.5 µl of 100 µM WT or 350 µM I38T mutant protein into a solution of 10 or 30 µM RO-7 followed by additional 3- or 2-µl injections. Experiments were carried out at 25°C. Results were analyzed using Origin software (OriginLab, Northampton, MA) provided by MicroCal. Binding constants (*K*_*D*_) were calculated from the average of three individual titrations by fitting the data to a single-site binding model using a nonlinear least-squares fitting algorithm.

### (ix) Statistical analysis.

The phenotypic data are presented as means with standard deviations (SD) from triplicate determinants in representative experiments or combined data from at least 3 to 6 independent experiments as indicated. The EC_50_ values were determined by nonlinear regression curve fitting using the log (inhibitor) versus response logistic equation (plaque assay) or Sigmoidal, 4PL (minireplicon assay) in GraphPad Prism 7.0 software.

### Accession number(s).

The Protein Data Bank IDs of the deposited crystal structures are 5VP8, 5VPT, 5VPX, 5VQN, and 5VRJ.
