# Author Correction for Jones et al., “Identification of the I38T PA Substitution as a Resistance Marker for Next-Generation Influenza Virus Endonuclease Inhibitors”

**DOI:** 10.1128/mBio.01868-18

**Published:** 2018-11-13

**Authors:** Jeremy C. Jones, Gyanendra Kumar, Subrata Barman, Isabel Najera, Stephen W. White, Richard J. Webby, Elena A. Govorkova

**Affiliations:** a Department of Infectious Diseases, St. Jude Children’s Research Hospital, Memphis, Tennessee, USA; b Department of Structural Biology, St. Jude Children’s Research Hospital, Memphis, Tennessee, USA; c Independent consultant and collaborator with St. Jude Children’s Research Hospital, Memphis, Tennessee, USA

## AUTHOR CORRECTION

Volume 9, issue 2, e00430-18, 2018, https://doi.org/10.1128/mBio.00430-18. We correct the following error in our publication. The primary focus of the work is on the description of antiviral resistance-associated markers in the influenza PA endonuclease domain (PA_N_, amino acids 1 to ≈200), which is responsible for the endonuclease activity of the virus. Figure S1 presents data from minigenome assays that measure cumulative viral polymerase activity with and without the endonuclease inhibitor RO-7. The construct used in these assays contained the I38T resistance substitution in the PA plasmid. After further sequencing of this construct, two additional unexpected changes, P224S and P295L, both outside the endonuclease domain, were detected. Neither mutation was described in the original manuscript.

In a summary of four independent experiments, we have subsequently determined that P224S/P295L in combination with I38T lower polymerase activity approximately 1.7-fold with the CA/04 virus. When only I38T is present, a statistically insignificant decrease was observed, suggesting that P224S and P295L do negatively impact polymerase activity, albeit mildly. P224S and P295L were not present in the PR/8 PA plasmid.

Regardless of the impact on output from minigenome assays, the main conclusions of the work, that I38T is a critical determinant of endonuclease inhibitor resistance, remain unaltered. Importantly, the presence of P224S/P295L in conjunction with I38T does not impact resistance to RO-7. In the presence of 250 nM RO-7, the CA/04 constructs containing I38T, P224S, and P295L retain 71.6% of normalized polymerase activity compared to 72.6% of the I38T-alone plasmid. Further, we have confirmed that the P224S/P295L substitutions are not present in either the WT or the reverse genetics CA/04 or PR/8 viruses generated in this study and used to evaluate RO-7-resistant viruses (listed in Table 1).

Figure S1 and its legend have been replaced online.

