# Supplementary figures and images for: Identification of the I38T PA Substitution as a Resistance Marker for Next-Generation Influenza Virus Endonuclease Inhibitors
Source: mBio. 2018 Apr 24;9(2):e00430-18. doi: 10.1128/mBio.00430-18 (PMC5915737; doi:10.1128/mBio.00430-18)

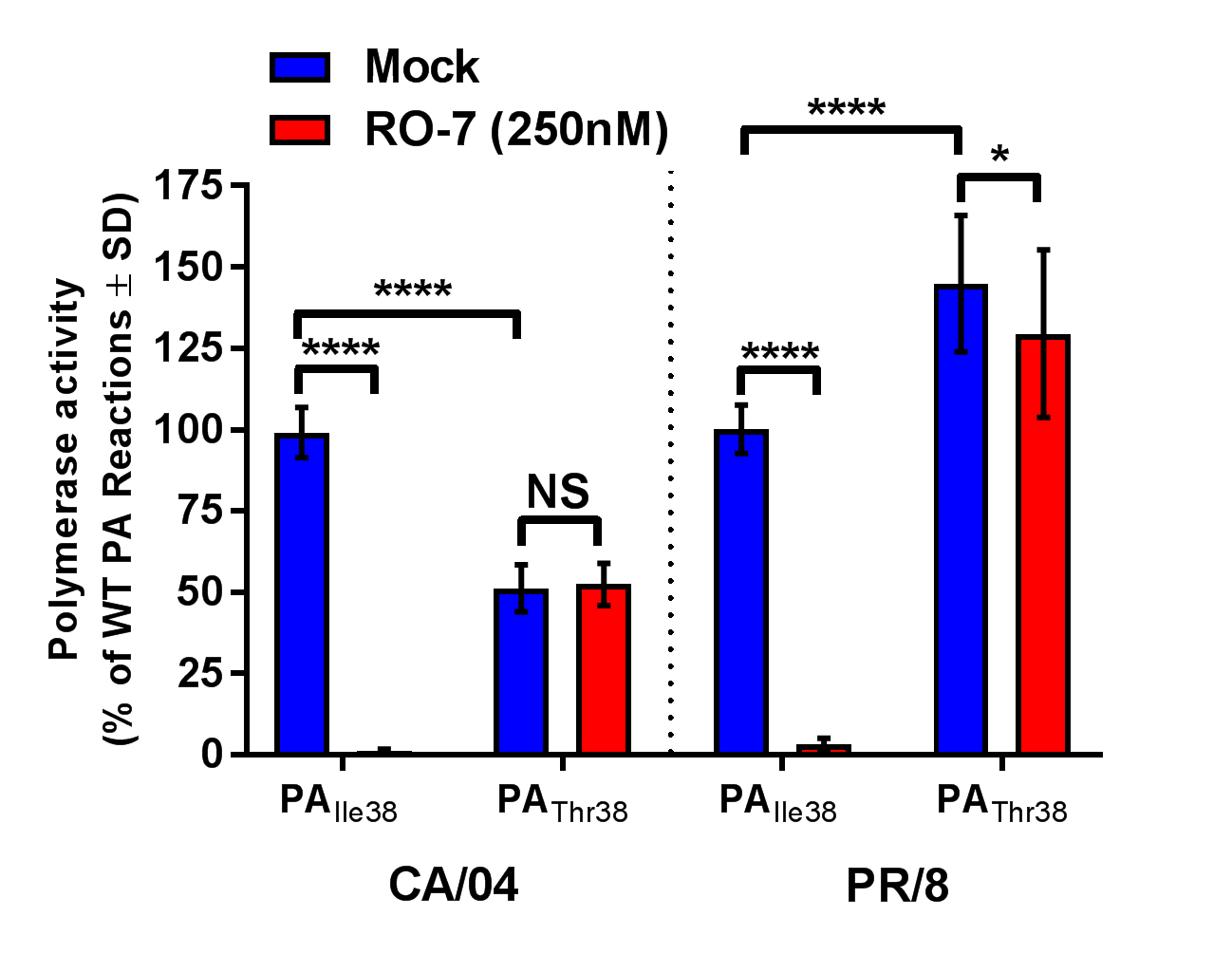

Supplement: FIG S1 [file mbo002183845sf1.tif]

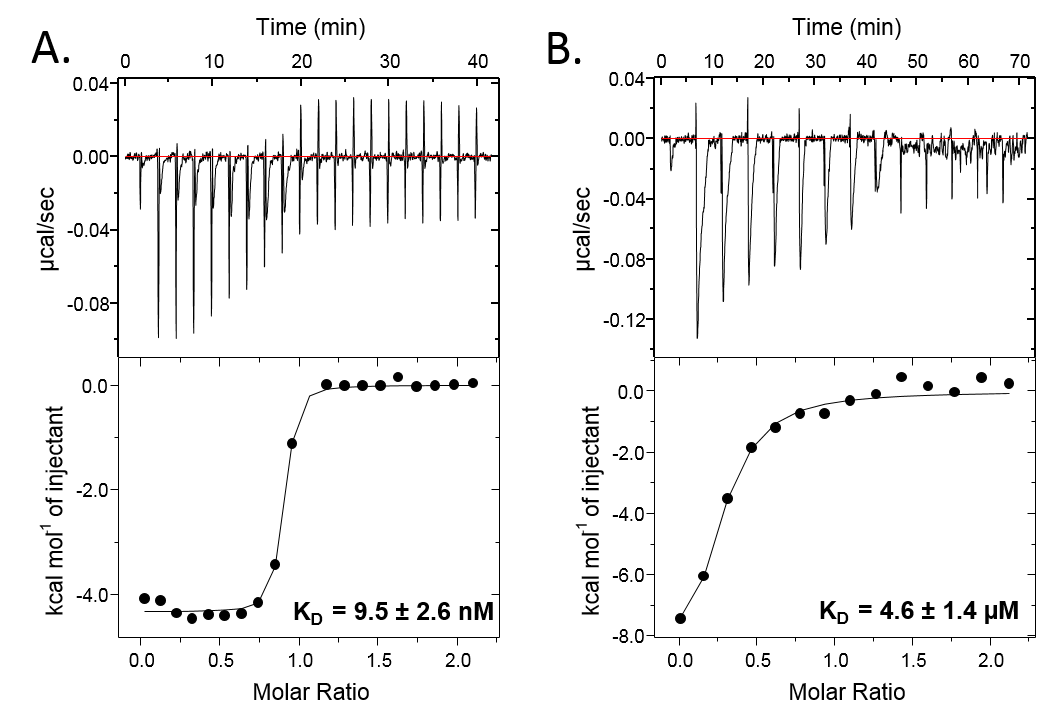

Supplement: FIG S2 [file mbo002183845sf2.tif]

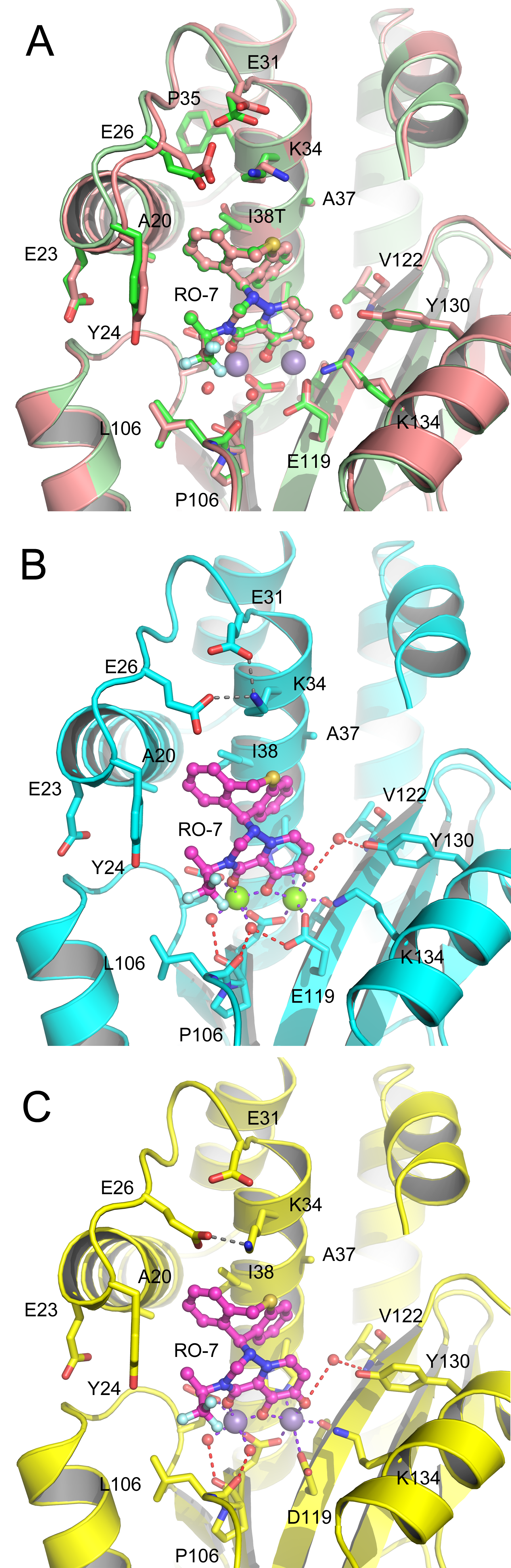

Supplement: FIG S3 [file mbo002183845sf3.tif]
