# Supplementary material for: Identification of the I38T PA Substitution as a Resistance Marker for Next-Generation Influenza Virus Endonuclease Inhibitors
Source: mBio. 2018 Apr 24;9(2):e00430-18. doi: 10.1128/mBio.00430-18 (PMC5915737; doi:10.1128/mBio.00430-18)
Supplement: TABLE S1 [file mbo002183845st1.docx]

**SUPPLEMENTAL TABLE 1.** **X-ray** **data collection and refinement statistics I.**

|  | WT PA_N_-Mn^2+^-RO-7  (PDB ID: 5VPT) | PA_N_(I38T)-Mn^2+^-RO-7  (PDB ID: 5VPX) | PA_N_(I38T)-Mn^2+^  (PDB ID: 5VP8) |
| --- | --- | --- | --- |
| **Data collection** |  |  |  |
| Wavelength (Å) | 1.000 | 1.000 | 1.000 |
| Space group | I422 | I422 | I422 |
| Cell dimensions |  |  |  |
| *a*, *b*, *c* (Å) | 89.88 89.88 133.73 | 90.67 90.67 134.16 | 90.1 90.1 134.12 |
| *α, β, γ* (°) | 90 90 90 | 90 90 90 | 90 90 90 |
| Resolution (Å) | 50 - 2.09 (2.17 - 2.09)^a^ | 50 - 2.30 (2.38 - 2.30) | 50 - 2.2 (2.28 - 2.2) |
| *R*_meas,_ | 0.050 (0.658) | 0.085 (0.834) | 0.090 (0.978) |
| *I/*σ(*I*) | 38.4 (2.0) | 35.92 (1.67) | 35.38 (1.53) |
| Completeness (%) | 97.7 (83.3) | 99.2 (92.9) | 99.23 (94.58) |
| Redundancy | 7.9 (5.3) | 10.9 (6.7) | 12.5 (8.3) |
|  |  |  |  |
| **Refinement** |  |  |  |
| Resolution (Å) | 38.49 - 2.09 (2.17 - 2.09) | 38.82 - 2.30 (2.38 - 2.30) | 38.59 - 2.2 (2.28 - 2.2) |
| No. reflections | 16175 (1320) | 12653 (1167) | 14287 (1325) |
| *R*_work_ / *R*_free_ | 0.20 / 0.22 | 0.19 / 0.22 | 0.20 / 0.24 |
| No. atoms | 1506 | 1497 | 1509 |
| Protein | 1440 | 1443 | 1443 |
| Ligand/ion | 41 | 46 | 45 |
| Water | 25 | 8 | 21 |
| *B* factors |  |  |  |
| Protein | 68.28 | 76.59 | 65.11 |
| Ligand/ion | 61.01 | 78.59 | 78.84 |
| Water | 62.51 | 61.03 | 57.36 |
| R.m.s. deviations |  |  |  |
| Bond lengths (Å) | 0.007 | 0.008 | 0.026 |
| Bond angles (°)  Ramachandran Plot  Favoured (%)  Allowed (%)  Outliers (%) | 0.88  94.35  5.65  0.00 | 0.87  93.79  6.21  0.00 | 2.35  95.48  4.52  0.00 |
|  |  |  |  |

^a^ Values in parentheses are for highest-resolution shell.
