# Supplementary material for: Identification of the I38T PA Substitution as a Resistance Marker for Next-Generation Influenza Virus Endonuclease Inhibitors
Source: mBio. 2018 Apr 24;9(2):e00430-18. doi: 10.1128/mBio.00430-18 (PMC5915737; doi:10.1128/mBio.00430-18)
Supplement: TABLE S2 [file mbo002183845st2.docx]

**SUPPLEMENTAL TABLE 2.** **X-ray** **data collection and refinement statistics II.**

|  | WT PA_N_-Mg^2+^-RO-7  (PDB ID: 5VRJ) | PA_N_(E119D)- Mn^2+^-RO-7  (PDB ID: 5VQN) |
| --- | --- | --- |
| **Data collection** |  |  |
| Wavelength (Å) | 1.000 | 1.000 |
| Space group | I422 | I422 |
| Cell dimensions |  |  |
| *a*, *b*, *c* (Å) | 90.92 90.92 133.91 | 89.85 89.85 133.26 |
| *α, β, γ* (°) | 90 90 90 | 90 90 90 |
| Resolution (Å) | 50 - 2.30 (2.38 - 2.30) ^a^ | 50 - 2.00 (2.07 - 2.00) |
| *R*_meas,_ | 0.087 (0.931) | 0.095 (0.954) |
| *I/*σ(*I*) | 29.39 (1.4) | 29.6 (1.04) |
| Completeness (%) | 98.91 (90.42) | 91.6 (53.4) |
| Redundancy | 7.9 (4.7) | 8.5 (4.6) |
|  |  |  |
| **Refinement** |  |  |
| Resolution (Å) | 37.61 - 2.30 (2.38 - 2.30) | 37.25 - 2.00 (2.07 - 2.00) |
| No. reflections | 12677 (1132) | 17150 (973) |
| *R*_work_ / *R*_free_ | 0.20 / 0.25 | 0.20 / 0.24 |
| No. atoms | 1482 | 1503 |
| Protein | 1438 | 1436 |
| Ligand/ion | 41 | 46 |
| Water | 3 | 21 |
| *B* factors |  |  |
| Protein | 75.98 | 64.03 |
| Ligand/ion | 72.52 | 68.10 |
| Water | 63.39 | 59.74 |
| R.m.s. deviations |  |  |
| Bond lengths (Å) | 0.008 | 0.005 |
| Bond angles (°)  Ramachandran Plot  Favoured (%)  Allowed (%)  Outliers (%) | 0.83  95.48  4.52  0.00 | 0.63  96.61  3.39  0.00 |
|  |  |  |

^a^ Values in parentheses are for highest-resolution shell.
