# Supplementary material for: Identification of the I38T PA Substitution as a Resistance Marker for Next-Generation Influenza Virus Endonuclease Inhibitors
Source: mBio. 2018 Apr 24;9(2):e00430-18. doi: 10.1128/mBio.00430-18 (PMC5915737; doi:10.1128/mBio.00430-18)
Supplement: TABLE S3 [file mbo002183845st3.docx]

| SUPPLEMENTAL TABLE 3. Amino acid changes in RO-7 resistant and sensitive influenza A (H1N1) viruses | | | | | | | | | | | | | | | | | | | |
| --- | --- | --- | --- | --- | --- | --- | --- | --- | --- | --- | --- | --- | --- | --- | --- | --- | --- | --- | --- |
| Influenza A virus | Passage description | RO-7 passaged | Passage no. | PB2 | | PB1 | | PA | | | | HA | | NP | NA | M1 | M2 | NS1 | NEP |
|  |  |  |  | 504*^a^* | 702 | 228 | 636 | 38 | 199 | 347 | 349 | 73 | 345 | - | - | - | - | 78 | - |
| A/California/04/2009 (H1N1)pdm09 | Input virus | No | 0 | V*^b^* | K | T | E | I | E | D | E | N | G | - | - | - | - | R | - |
|  | No drug virus passage*^c^* | No | 16 | - | - | - | - | - | - | - | - | - | - | - | - | - | - | - | - |
|  | Mutant stability | Yes | S5 | - | - | - | K | T | K | - | G | - | - | - | - | - | - | - | - |
|  |  |  |  | 504 | 702 | 228 | 636 | 38 | 199 | 347 | 349 | 73 | 345 | - | - | - | - | 78 |  |
| A/Puerto Rico/8/1934 (H1N1) | Input virus | No | 0 | I | K | T | E | I | E | D | E | N | L | - | - | - | - | K | - |
|  | No drug virus passage | No | 16 | - | - | - | - | - | - | - | - | - | - | - | - | - | - | - | - |
|  | Mutant stability | Yes | S5 | V | R | A | - | T | G | N | - | D | I | - | - | - | - | E | - |

*^a^*Amino acid position.

*^b^*Amino acid identity determined by Sanger sequencing.

*^c^*Truncated segments corresponding to amino acid changes from input virus were sequenced.

(-) No amino acid change from sensitive virus.
